# Editorial: CD4^+^ T Cells in Cancer Immunotherapies

**DOI:** 10.3389/fimmu.2021.737615

**Published:** 2021-09-06

**Authors:** José M. González-Navajas, Eyad Elkord, Jongdae Lee

**Affiliations:** ^1^Alicante Institute for Health and Biomedical Research (ISABIAL), Alicante, Spain; ^2^Department of Pharmacology, University Miguel Hernández (UMH), Elche, Spain; ^3^School of Science, Engineering and Environment, University of Salford, Manchester, United Kingdom; ^4^School of Basic Medical Sciences and State Key Laboratory of Respiratory Disease, Guangzhou Medical University, Guangzhou, China

**Keywords:** CD4, immunotherapy, immune checkpoint inhibitor (ICI), cancer, T lymphocyte

In the last 20 years, remarkable advances in the field of immunotherapy have led to new therapeutic options for patients with a wide range of cancer types. Among the different immunotherapy approaches, immune checkpoint inhibition (ICI) therapy and chimeric antigen receptor (CAR) T cells have arguably shown the greatest potential. All immunotherapies share a common goal, which is to activate T lymphocytes and reinvigorate immune surveillance against cancer. While the role of CD8^+^ T lymphocytes and other cytotoxic cells in tumor immunology has been extensively studied, the importance of CD4^+^ T helper (Th) cells has been traditionally underestimated. In this special Research Topic, the relationship between different CD4^+^ T cell subsets and the efficacy of current cancer immunotherapies has been highlighted by several reviews and an original article.

A review article by Zuazo et al. puts systemic CD4^+^ T cells in the spotlight of PD1/PD-L1 blocking therapies, with a special focus on non-small cell lung cancer (NSCLC). Rather than changes in the tumor microenvironment, the authors bring attention to the often overlooked but significant impact that these therapies exert over systemic immunity. Pharmacodynamic changes in circulating PD1^+^ CD8^+^ T cells after anti-PD-1 treatment have been correlated with clinical response (1) and recent evidence suggests that these changes, in turn, may depend on systemic CD4^+^ cells (2). Hence, Zuazo et al. argue that targeting systemic CD4 immunity might be an important element of PD1-based therapies and propose that pre-treatment levels of specific CD4^+^ T cell memory subsets in the periphery are correlated with response in NSCLC.

DeRogatis et al. review the role of PSGL-1 (P-selectin glycoprotein ligand-1) as an immune checkpoint of CD4^+^ T cells. PSGL-1 is highly expressed in CD4^+^ T cells and binds to multiple proteins including P-selectin and VISTA. *Psgl-1^-/-^* mice mount a potent CD4^+^ and CD8^+^ T cell response against melanoma tumors compared to control mice. It is proposed that they energize CD8^+^ T cells by targeting PSGL-1 in CD4^+^ T cells or target PSGL-1 along with anti-PD-1/PD-L1 and/or anti-CTLA-4.

Successful ICI therapy requires appropriate effector Th responses. The diverse and sometimes opposing roles of effector Th cells, in particular Th1, Th9, and Th17 cells, has also been reviewed in an article by Lee et al. The process of Th cell differentiation allows for considerable plasticity among different Th subpopulations, which may be exploited to increase the efficacy of ICI therapy. For this strategy to work, more data are necessary to clearly determine what type of Th subpopulation is beneficial in each particular cancer type, and then identify suitable approaches to convert other Th populations into that population. In a related article, Basu et al. discuss ways to exploit the opposing actions of Th cell subtypes to amplify immunotherapy success. They extensively document the roles that dendritic cells (DCs) play during Th differentiation and CD8^+^ T cell functions and propose that combinatorial approaches can drive multiple Th subtypes to overcome potential obstacles to immunotherapy such as a hostile tumor microenvironment (TME), presence of inhibitory T cell populations and immune checkpoint receptors. Some of these approaches to regulating CD4^+^ T cell function in the TME might be based on epigenetic processes. Epigenetic regulation is involved in Th cell differentiation and plasticity both in homeostasis and in pathological conditions, such as inflammatory bowel disease (3). In this Research Topic, Renaude et al. discuss how epigenetic manipulation may induce a favorable immune context in the TME and improve the efficacy of immunotherapies, including adoptive cell transfer, anti-cancer vaccines, and ICI therapy.

While certain Th subpopulations can boost the anti-tumor immune response, one of the major hurdles of current cancer immunotherapies is the presence of regulatory T (Treg) cells in the TME. Treg cells not only accumulate in tumors but also show a higher immunosuppressive capacity than their non-intratumoral counterparts (4). González-Navajas et al. review current understanding of how Treg cells inhibit anti-cancer immunity and ICI therapy and discuss several strategies to specifically deplete intratumoral Tregs or to convert them into effector T cells without inducing systemic inflammation. Although important advances have been made in this direction, a precise characterization of Treg cell populations and identification of intratumoral Treg-specific targets in each cancer type are still needed. In this sense, an original article by Di Giorgio et al. reports a new molecular mechanism that controls Treg suppressive abilities. The authors show that loss of the transcription factor monocyte enhancement factor 2c (Mef2c) in Foxp3^+^ Treg cells switches on the transcription of histone deacetylase 9 (Hdac9), which in turn blocks Mef2d, a transcription factor that sustains Treg suppressive functions (5). Thus, Mef2c deletion led to the impairment of Treg cells *in vivo* and enhanced anti-tumor immunity in a syngeneic lung cancer model. Of note, Hdac9 is induced by Mef2d as part of a negative feedback loop (6). Thus, Di Giorgio et al. propose a mechanism whereby two members of the Mef2 family, Mef2c and Mef2d, coordinate to turn the expression of Hdac9 on and off and support Treg immune suppressive functions.

In conclusion, the papers included in this Research Topic highlight the distinct contribution of different CD4^+^ T cell populations to cancer immune surveillance and immunotherapy.

## Author Contributions

All authors contributed to the article and approved the submitted version.

## Conflict of Interest

The authors declare that the research was conducted in the absence of any commercial or financial relationships that could be construed as a potential conflict of interest.

## Publisher’s Note

All claims expressed in this article are solely those of the authors and do not necessarily represent those of their affiliated organizations, or those of the publisher, the editors and the reviewers. Any product that may be evaluated in this article, or claim that may be made by its manufacturer, is not guaranteed or endorsed by the publisher.
